# Reassessment of Growth and Exploitation of *Portunus trituberculatus* in Laizhou Bay: Legacy of Historical Overfishing

**DOI:** 10.3390/ani16132021

**Published:** 2026-07-02

**Authors:** Shihao Chen, Jilong Chen, Sihan Zhang, Fan Li, Xiaomin Zhang, Haixia Su

**Affiliations:** 1 College of Marine Living Resources Sciences and Management, Shanghai Ocean University, Shanghai 201306, China; csh07jkfd@163.com (S.C.); silentouhai@163.com (J.C.); zsh13539367331@126.com (S.Z.); 2 Shandong Provincial Key Laboratory of Restoration for Marine Ecology, Shandong Marine Resource and Environment Research Institute, Yantai 264006, China; fishery.lifan@hotmail.com; 3 Observation and Research Station of Laizhou Bay Marine Ecosystem, MNR, Yantai 264006, China

**Keywords:** *Portunus trituberculatus*, FiSAT II, growth parameters, fishing pressure, population resources, Laizhou Bay

## Abstract

To investigate growth trends of *Portunus trituberculatus* in Laizhou Bay, this study fitted the Von Bertalanffy growth model using FiSAT II based on carapace width frequency data from 2023 to 2025. Growth parameters and mortality coefficients were estimated, and natural mortality was evaluated using eight empirical formulas. A total of 2240 individuals (1117 females, 1123 males) were captured. The carapace width–weight relationships were *W* = 7.145 × 10^−5^*L*^2.9153^ (total), *W* = 1.208 × 10^−4^*L*^2.8051^ (females), and *W* = 3.209 × 10^−5^*L*^3.0809^ (males), indicating negative allometric growth in females and near-isometric growth in males. Asymptotic carapace widths (*L*_∞_) were 228.32, 227.23, and 215.25 mm; growth rates (*k*) were 0.43, 0.49, and 0.44; and the total mortalities (*Z*) were 1.41, 1.44, and 1.30 for total, female, and male, respectively. Recruitment occurred from April to August (spring–summer). Mean natural mortality (*M*) was 0.70, and exploitation rate (*E*) was 0.50. Compared with historical data, the current exploitation rate has decreased from the severely overexploited levels observed historically, approaching the commonly used MSY reference point of *E* = 0.50. This decline reflects the positive effects of summer fishing moratoriums and stock enhancement in reducing fishing pressure. However, this apparent stabilization in exploitation rate has not translated into biological recovery. Declining growth parameters and continued population decline indicate that the stock remains vulnerable, and current pressure may still exceed the ecosystem’s degraded carrying capacity. Future efforts should strengthen the monitoring and recovery of genetic diversity in *P. trituberculatus*, extend the study period of length–frequency data, and construct ecosystem models to assess the impacts of food competition and habitat changes, thereby providing a scientific basis for stock enhancement and resource assessment of *P. trituberculatus* in Laizhou Bay.

## Simple Summary

Fishery resources in coastal ecosystems are under increasing pressure from fishing and environmental change, making it essential to track population dynamics for effective management. The swimming crab *Portunus trituberculatus* is an economically valuable species in Laizhou Bay, China, where it supports important fisheries and stock enhancement programs. In this study, we analyzed the growth, mortality, and population status of this crab using recent survey data and modeling tools. Our results show that current fishing pressure has been reduced to a more sustainable level, largely due to summer fishing moratoriums and stock enhancement. However, despite these management efforts, key growth-related indicators have continued to decline, reflecting lasting effects from past overfishing and environmental degradation. These findings underscore the need for long-term recovery strategies, including genetic diversity monitoring, extended field surveys, and ecosystem-based models. This study provides practical scientific support for adaptive management and restoration of swimming crab resources in Laizhou Bay.

## 1. Introduction

*Portunus trituberculatus* (*Crustacea*, *Decapoda*, *Portunidae*, *Portunus*) is a large commercially important crustacean species in Chinese coastal waters [1], widely distributed across the Bohai Sea, Yellow Sea, East China Sea, and South China Sea. This crab has a short life cycle, high fecundity, and exhibits a distinct nocturnal habit, commonly inhabiting sandy–muddy seabeds at depths of 10–30 m in coastal waters. Due to differences in their life history traits and geographical conditions among sea regions, local populations of *P. trituberculatus* in China exhibit regional variations. Laizhou Bay, located in the southern Bohai Sea and northern Shandong Province, is one of the three major bays of the Bohai Sea. With its nutrient-rich waters and abundant prey availability, Laizhou Bay serves as a critical spawning ground and habitat for the Bohai Sea population of *P. trituberculatus* [2]. In recent years, increased fishing pressure and environmental pollution caused by coastal development have led to substantial fluctuations in the community structure of Laizhou Bay, accompanied by a decline in biodiversity indices, including the Shannon–Weaver diversity index (H′), species richness (D), and Pielou’s evenness index (J), indicating a trend toward community homogenization and reduced ecosystem complexity [3]. Concurrently, persistent pollution inputs from terrestrial sources, primarily industrial effluents, agricultural runoff, and domestic wastewater transported via inflowing rivers such as the Yellow River, Xiaoqing River, and Wei River, have further degraded the water quality and habitat conditions in the bay [4]. As a consequence, multiple fishery resources, including *P. trituberculatus*, have shown signs of population decline [5]. Since 2005, Shandong Province has implemented resource stock enhancement programs, with *P. trituberculatus* being the primary released species in the bay, playing a key role in energy flow and nutrient cycling within the Laizhou Bay ecosystem [6].

As a key commercial crustacean species in Laizhou Bay, *P. trituberculatus* plays a critical role in the study of fishery resource ecology, particularly regarding its biological parameters [7]. Wu et al. indicated that despite the overall decline in fishery resources in Laizhou Bay, *P. trituberculatus* still maintained a relatively high resource density during specific periods, and stock enhancement has sustained its population size to some extent [8]. Cong et al. systematically studied the community structure of *P. trituberculatus* in Laizhou Bay, and their results showed that stock enhancement and the summer fishing moratorium policy have played positive roles in the recovery of its resource abundance, although a gap remains compared with traditional historical levels [9]. Yang et al. estimated the growth parameters and mortality coefficients of *P. trituberculatus* in Laizhou Bay in 2012. Despite an exploitation rate (*E* = 0.684–0.712) well above the optimal level, the population retained a rapid growth coefficient (*k* = 1.50–1.60) and large asymptotic carapace width (*L*_∞_ = 210–241.5 mm). This combination of overexploitation and high growth potential suggested that the stock, although heavily fished, had not yet lost its capacity for recovery, indicating a certain potential for stock enhancement if the fishing pressure were reduced [7]. However, after more than a decade of environmental changes and the continuous expansion of stock enhancement efforts in the bay, the current growth status of *P. trituberculatus* in this area has not yet been reported.

The assessment and management of fishery resources rely on the timely understanding of population dynamics. Using FiSAT II software and survey data of *P. trituberculatus* collected in Laizhou Bay from 2023 to 2025, this study reconstructed the growth model of *P. trituberculatus* in the bay over the past three years, updating key parameters including the asymptotic carapace width (*L*_∞_), growth coefficient (*k*), total mortality coefficient (*Z*), and exploitation rate (*E*). By comparing these results with historical data, this study aims to reveal the changing trends in population growth characteristics, evaluate the effectiveness of current management measures, and provide a scientific basis for future stock enhancement and resource assessment of *P. trituberculatus*.

## 2. Materials and Methods

### 2.1. Sample Collection

**Figure 1:**
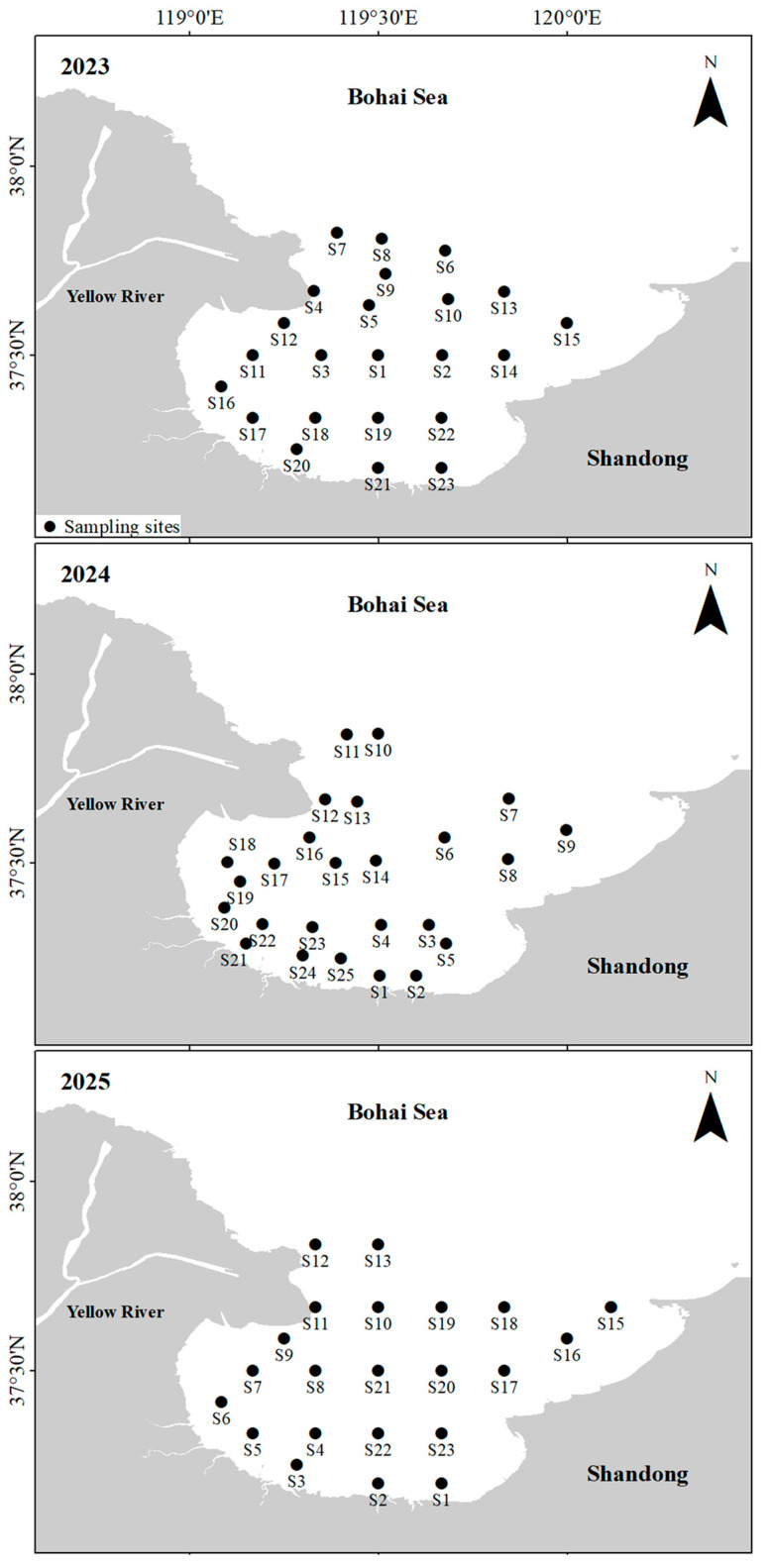
Location of the sampling stations in Laizhou Bay from 2023 to 2025.

The data used in this study were obtained from bottom trawl surveys conducted by a single vessel in Laizhou Bay (119°05′–120°00′ E, 37°12′–38°05′ N) from May to August each year between 2023 and 2025. Survey stations for the 3 years are shown in Figure 1. A total of 2240 *P. trituberculatus* individuals were collected and subjected to biological measurements, with carapace width and body weight measured to the nearest 1 mm and 0.1 g, respectively. The survey vessel had a power of 260 kW. The sampling gear was a bottom trawl operated by a single vessel, with a net mouth circumference of 30.6 m and a codend mesh size of 20 mm. Trawling was conducted for one hour per station at a towing speed of 3.0 knots, with the net mouth width being approximately 8 m during towing.

It should be noted that all captured specimens were treated as a single population of *P. trituberculatus*. Distinguishing between naturally recruited and hatchery-released individuals would require systematic sampling at fixed stations before and after release, followed by quantitative analysis. The data used in this study were derived from routine fishery resource surveys, without paired sampling before and after stocking. Consequently, length–frequency analyses based on mixed-population data could not effectively differentiate between the two sources. This limitation should be fully considered when interpreting the recruitment patterns and resource assessment results.

### 2.2. Estimation of Growth Parameters

The relationship between the carapace width and body weight of *P. trituberculatus* was fitted using a power function as follows:

(1) $$W\;=\;a\;\times \;L^{b\;}$$

where *W* is the body weight (g), *L* is the carapace width (mm), *a* is the condition factor, and *b* is the power exponent.

Based on the measured carapace width and body weight data, a carapace width frequency dataset was constructed with a class interval of 10 mm [10]. The ELEFAN I (Electronic Length Frequency Analysis I) method implemented in FiSAT II software (version 1.2.2; FAO-ICLARM, 2005) was used to fit the Von Bertalanffy growth function (VBGF) and estimate the growth parameters *L*_∞_ (asymptotic carapace width) and *k* (growth coefficient) for *P. trituberculatus* in Laizhou Bay. The VBGF is expressed as follows:

(2) $$L_{t}\;={\;L}_{\infty }\left[1-e^{-K(t-t_{0})}\right]$$

(3) $$W_{t}={\;W}_{\infty }{\left[1-e^{-K(t-t_{0})}\right]}^{b}$$

where *L_t_* and *W_t_* are the carapace width and body weight at time *t*, respectively, and *t*_0_ is the theoretical age at which growth begins. The parameter *t*_0_ was estimated using Pauly’s empirical formula:

(4) $$ln(-t_{0})\;=\;-0.3922-0.2752lnL_{\infty }-1.308lnK$$

The goodness-of-fit of the biological parameters was evaluated using the Score index proposed by Pauly et al. [11]. The Score index ranges from 0 to 1, serving as an estimate of the goodness-of-fit. To ensure reproducibility, we performed a *k*-scan across a range of *k* values (0.10–2.00 year^−1^, at 0.05 increments) with an initial *L*_∞_ estimate based on the maximum observed carapace width. The *k* yielding the highest Score index was selected as the starting point, followed by a response surface analysis that varied *L*_∞_ (±20%) and *k* (±50%) simultaneously to identify the global optimum combination. By iteratively adjusting the initial values of *L*_∞_ and *k*, the optimal values that yield the maximum Score index while remaining biologically acceptable were determined. This index was simultaneously calculated within the FiSAT II software using the following formula:

(5) $$\mathrm{Score}\;=\;10^{\mathrm{ESP}/\mathrm{ASP}}/10$$

where *ESP* (Explained Sum of Peaks) represents the sum of theoretical peaks, and *ASP* (Available Sum of Peaks) represents the maximum achievable sum of peaks from the curve.

### 2.3. Estimation of Death Coefficient

The total mortality coefficient (*Z*) was estimated using the length-converted catch curve method implemented in FiSAT II software. The formula is expressed as follows:

(6) $$Ln(N_{t}/∆t)\;=\;a\;+\;b\;\times \;t$$

where *N_t_* is the proportion of individuals in the carapace width group at age *t* relative to the total sample number, Δ*t* is the time required for growth from the lower limit to the upper limit of the corresponding length class, *t* is the median age of the corresponding length class, *a* = *lnN*_0_, and −*b* = *Z*, which represents the estimated total mortality coefficient.

The natural mortality coefficient (*M*) is generally difficult to estimate. Previous studies have mostly relied on Pauly’s empirical formula [11], while the accuracy of other related methods remains inconclusive. Brodziak et al. proposed averaging the results obtained from multiple estimation methods to reduce uncertainty in the estimates [12]. In this study, eight commonly used empirical methods for estimating *M* (Table 1) were referenced, and the average of the results from these empirical formulas was used as a reference value for the natural mortality coefficient of *P. trituberculatus*. The differences in resource utilization of the crab based on various *M* estimates were compared to evaluate the growth characteristics of *P. trituberculatus* in Laizhou Bay. In Table 1, *T* represents the annual mean water temperature of the habitat, which was set as 12 °C for Laizhou Bay [13]; *T_max_* was defined based on the theoretical maximum age of *P. trituberculatus*, which was set as 3 years [14]. The fishing mortality coefficient (*F*) was calculated as the difference between total mortality and natural mortality, *F* = *Z* − *M*. The exploitation rate (*E*) was defined as the ratio of the fishing mortality coefficient to the total mortality coefficient, *E* = *F*/*Z*.

### 2.4. Population Recruitment Pattern and Resource Utilization

The carapace width frequency data were imported into FiSAT II software. Using the Recruitment Patterns module, the asymptotic carapace width (*L*_∞_), growth curvature (*k*), and theoretical age at birth (*t*_0_) were input to estimate the annual population recruitment period of *P. trituberculatus* in Laizhou Bay, thereby determining the population recruitment pattern.

A length-structured virtual population analysis (VPA) was performed on the catch numbers, population numbers, and biomass of *P. trituberculatus*. The formula is expressed as follows:

(7) $$N_{L+\Delta L}/C_{L}\;=\;Z_{L}e^{-\mathrm{ZL}\Delta t}/F_{L}\left(1-e^{-\mathrm{ZL}\Delta t}\right)$$

where *N_L+_*_Δ*L*_ is the population number of the length class from *L* to *L* + Δ*L*, *C_L_* is the catch number of length class *L*, and *F_L_* and *Z_L_* are the fishing mortality coefficient and total mortality coefficient for length class *L*, respectively, which were calculated using the growth equation.

The estimation of yield per recruit and biomass per recruit was performed using the Beverton and Holt Y/R Analysis module in FiSAT II software, based on the following formulas:

(8) $$Y^{\prime }/R\;=\;EU^{M/K}\times \left(1-\frac{3U}{1+m}+\frac{3U^{2}}{1+2m}-\frac{U^{3}}{1+3U}\right)$$

(9) $$B^{\prime }/R=\frac{Y^{\prime }/R}{F}$$

where *U* = 1 − (*L_C_*/*L*_∞_); m = 1 − *E_M_*/*K* = *K*/*Z*; *Y*′/*R* is the relative yield per recruit (g); *B*′/*R* is the relative biomass per recruit; *E* is the exploitation rate; *L_c_* is the mean selection carapace length, which was estimated as the carapace width at 50% retention, derived from the cumulative retention curve (based on the ratio of observed to expected values of unused catch-curve points) under the knife-edge selection assumption in FiSAT II; *L*_∞_ is the asymptotic carapace width; *M* is the natural mortality coefficient; *k* is the growth coefficient; *Z* is the total mortality coefficient; and *F* is the fishing mortality coefficient.

## 3. Results

### 3.1. Sex Ratio and Size Distribution

**Figure 2:**
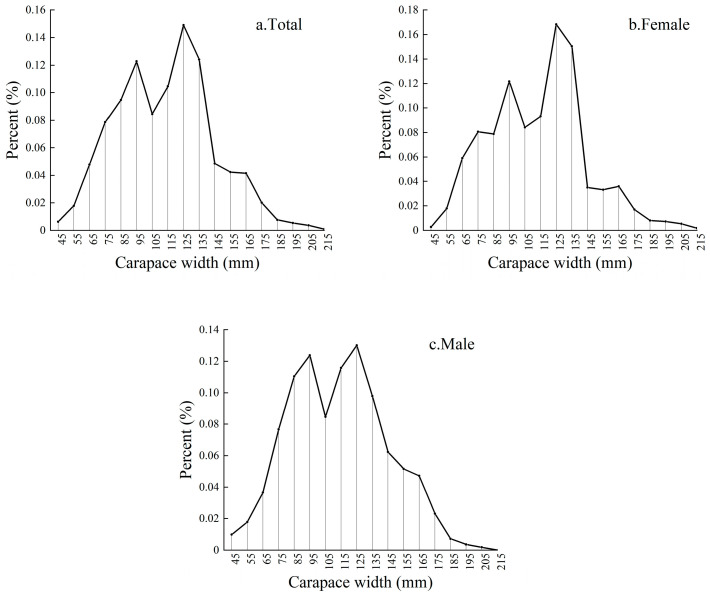
Frequency distribution of carapace width of *P. trituberculatus* in the Laizhou Bay. (**a**) Female swimming crabs; (**b**) male swimming crabs; and (**c**) total swimming crabs.

Based on the fishery resource surveys conducted in Laizhou Bay from 2023 to 2025, a total of 2240 individuals of *P. trituberculatus* were collected and measured, with a total weight of 172.09 kg. A total of 1117 females were collected, with a total weight of 84.31 kg and a carapace width ranging from 40 to 211 mm, of which individuals measuring 55–175 mm accounted for 97.49% of the total female count. A total of 1123 males were collected, with a total weight of 87.78 kg and a carapace width ranging from 35 to 205 mm, of which individuals measuring 55–175 mm accounted for 97.94% of the total male count. The female-to-male sex ratio was 0.99 and the chi-square test revealed no significant difference in the numbers of females and males (*p* > 0.05) (Figure 2).

### 3.2. Growth Equation Establishment

**Figure 3:**
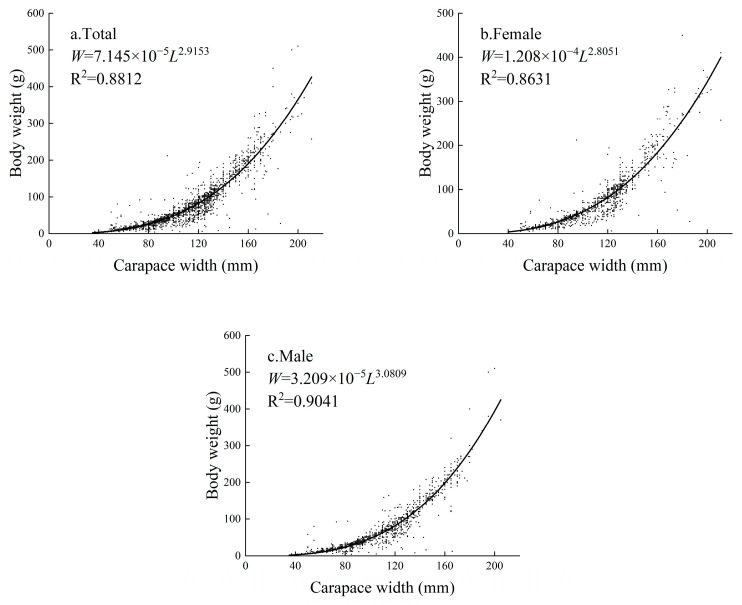
Relation curves between carapace width and body weight of *P. trituberculatus* in the Laizhou Bay. (**a**) Female swimming crabs; (**b**) male swimming crabs; and (**c**) total swimming crabs.

A power function curve was fitted to the carapace width and body weight data of the 2240 captured *P. trituberculatus* individuals (Figure 3). The fitted result was *W* = 7.145 × 10^−5^*L*^2.9153^ (R^2^ = 0.8812), with the power exponent *b* = 2.9153, indicating negative allometric growth (*p* < 0.05). The carapace width–body weight relationships for female and male individuals were *W* = 1.208 × 10^−4^*L*^2.8051^ (R^2^ = 0.8631) and *W* = 3.209 × 10^−5^*L*^3.0809^ (R^2^ = 0.9041), respectively. Female *P. trituberculatus* exhibited negative allometric growth (*p* < 0.05), whereas male individuals exhibited near-isometric growth (*p* > 0.05).

A carapace width frequency dataset for *P. trituberculatus* was constructed with a class interval of 10 mm (Pauly 1980 [11]). The ELEFAN I module in FiSAT II software was used to estimate the growth rate (*k*) and asymptotic carapace width (*L*_∞_) for both female and male individuals, and Pauly’s empirical formula was applied to estimate the theoretical age at which growth begins (*t*_0_). The results showed that for the total population, the *L*_∞_ was 228.32 mm, the *k* was 0.43, and the *t*_0_ was −0.46. For female crabs, the *L*_∞_ was 227.23 mm, the *k* was 0.49, and the *t*_0_ was −0.39. For male crabs, the *L*_∞_ was 215.25 mm, the *k* was 0.44, and the *t*_0_ was −0.45. The *k* of female crabs was higher than that of males, indicating faster growth in females. The specific growth equations for *P. trituberculatus* are as follows, for the total population: *L_t_* = 228.32[1 − *e*^−0.43(*t*+0.46)^], *W_t_* = 593.04[1 − *e*^−0.43(*t*+0.46)^]^2.9153^; for female crabs: *L_t_* = 227.23[1 − *e*^−0.49(*t*+0.39)^], *W_t_* = 493.10[1 − *e*^−0.49(*t*+0.39)^]^2.805^; and for male crabs: *L_t_* = 215.25[1 − *e*^−0.44(*t*+0.45)^], *W_t_* = 564.08[1 − *e*^−0.44(*t*+0.45)^]^3.0809^.

### 3.3. Mortality Coefficients and Mean Selection Carapace Width

**Figure 4:**
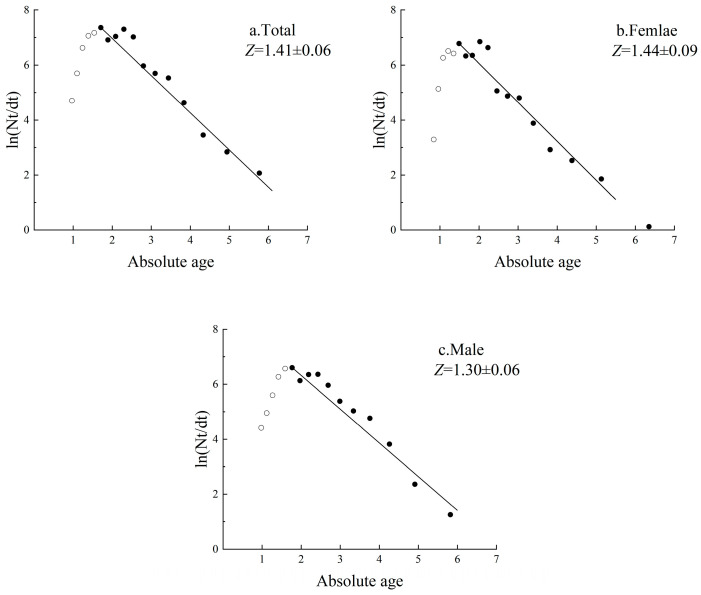
The length-converted catch curves of *P. trituberculatus* in Laizhou Bay. (**a**) Female swimming crabs; (**b**) male swimming crabs; and (**c**) total swimming crabs.

**Figure 5:**
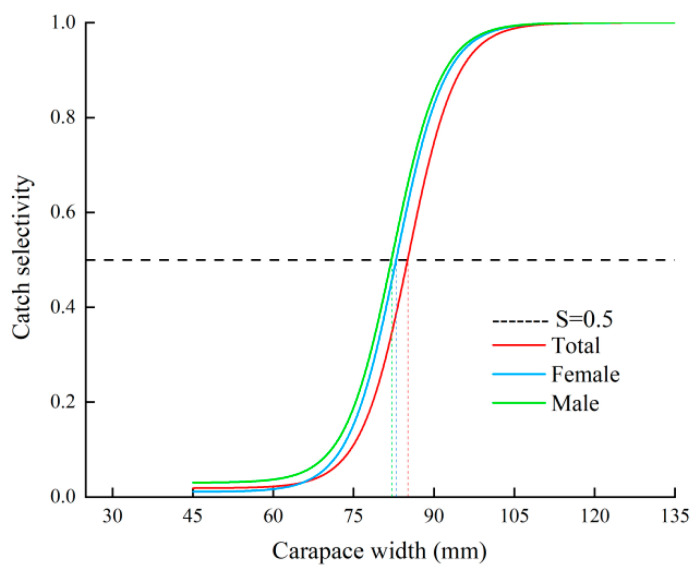
The catch selectivity curve of *P. trituberculatus* in Laizhou Bay.

The total mortality coefficient (*Z*) of *P. trituberculatus* was estimated using the length-converted catch curve method implemented in FiSAT II software. In the analysis, only data points corresponding to individuals that had entered the fully recruited age classes were selected for linear regression fitting (Figure 4). The results showed that the total mortality coefficients were *Z* = 1.41 for the total population, *Z* = 1.44 for females, and *Z* = 1.30 for males. In the length-converted catch curves described above, some observation points were not utilized in the fitted relationship. A fishing selectivity curve was constructed based on the cumulative ratio of observed to expected values of the unutilized points. The carapace width at which individuals had a 50% probability of being retained (*S* = 0.5) was used to define the size at first capture. The resulting estimates were as follows: 85.19 mm for the total population, 82.98 mm for females, and 82.19 mm for males (Figure 5).

In this study, the natural mortality coefficient (*M*) of *P. trituberculatus* was estimated using eight empirical methods shown in Table 1, yielding a range of 0.39–1.00/year, with uncertainty present in the estimates (Table 2). Methods 3 and 8 were excluded because they produced *M* values greater than the total mortality coefficient (*Z*) for the total and male populations, and yielded impractically low estimates for females, all of which did not align with theoretical expectations. Among the remaining methods, the lowest natural mortality coefficient (*M_min_* = 0.39) was obtained using Pauly’s empirical formula for the total population, corresponding to *F* = 1.02 and *E* = 0.72. Methods 2, 4, and 6 produced consistent results (*M* = 0.65), with *F* = 0.76 and *E* = 0.54. Method 5 yielded the highest natural mortality coefficient (*M_max_* = 1.00), corresponding to *F* = 0.41 and *E* = 0.29. The mean reference value obtained by averaging multiple estimation methods was *M_mean_* = 0.70, with *F* = 0.71 and *E* = 0.50. Based on this averaging approach, the natural mortality coefficients for females and males were estimated as *M* = 0.87 for females and *M* = 0.71 for males, respectively; the corresponding fishing mortality coefficients were *F* = 0.57 for females and *F* = 0.59 for males, and the exploitation rates were *E* = 0.40 for females and *E* = 0.45 for males.

### 3.4. Recruitment Pattern of P. trituberculatus

**Figure 6:**
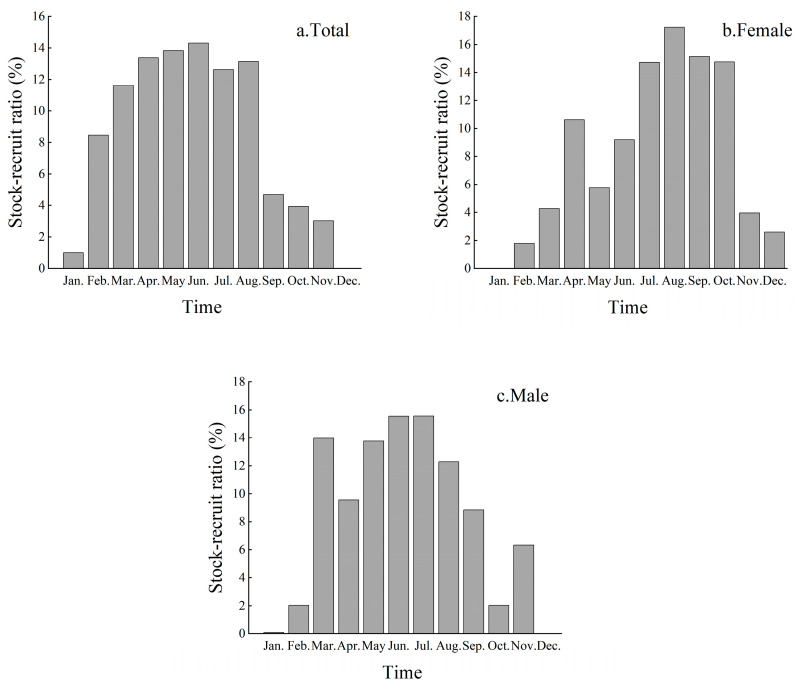
Recruitment pattern of *P. trituberculatus* in Laizhou Bay. (**a**) Female swimming crabs; (**b**) male swimming crabs; and (**c**) total swimming crabs.

From 2023 to 2025, the recruitment patterns of the total population and female *P. trituberculatus* in Laizhou Bay exhibited unimodal and continuous characteristics. In contrast, male crabs showed similar recruitment magnitudes in June and July, displaying a bimodal tendency. Overall, the three-year recruitment pattern of *P. trituberculatus* was characterized as a continuous spring–summer recruitment pattern, with the main recruitment period occurring from April to August (Figure 6).

### 3.5. Estimation of Population Biomass and Relative Yield per Recruit

**Figure 7:**
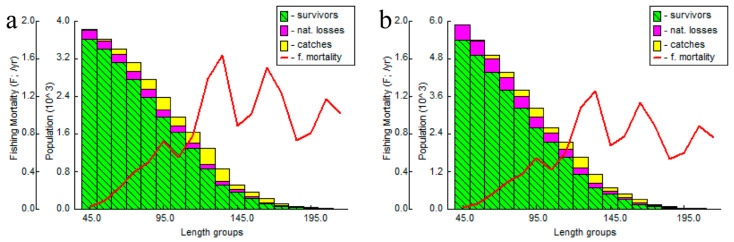

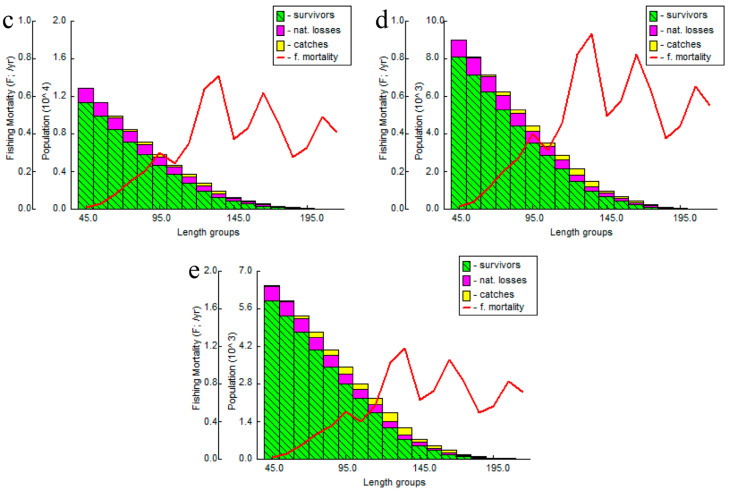
Survivors, losses, catches and fishing mortality curves of different carapace width classes of *P. trituberculatus* based on different natural mortality (*M*) estimation methods. (**a**) *M* = 0.39; (**b**) *M* = 0.65; (**c**) *M* = 1; (**d**) *M* = 0.86; (**e**) *M* = *M_mean_* = 0.7.

As shown in Figure 7, population abundance and biomass exhibited a clear positive dependence on the assumed value of natural mortality (*M*). Across the range of *M* examined (0.39 to 1.00), abundance varied from 3.59 × 10^8^ to 9.82 × 10^8^ ind., while biomass ranged from 2.81 × 10^4^ to 6.72 × 10^4^ t. Intermediate *M* values yielded intermediate outcomes: at *M* = 0.65 and 0.86, abundance was estimated at 5.05 × 10^8^ and 7.20 × 10^8^ ind., with a corresponding biomass of 3.71 × 10^4^ and 5.11 × 10^4^ t. Using the mean reference value (*M_mean_* = 0.70), the resulting abundance and biomass were 5.45 × 10^8^ ind. And 3.97 × 10^4^ t, respectively.

**Figure 8:**
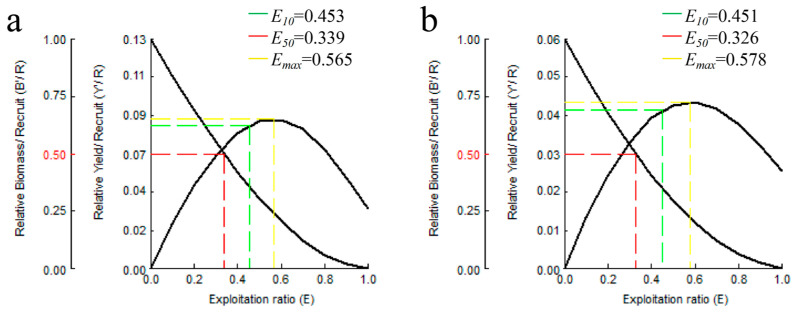

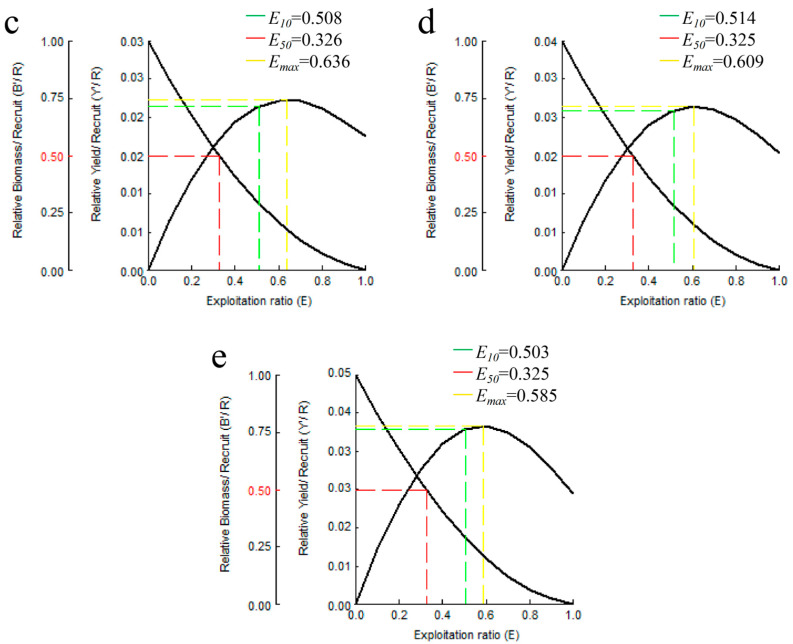
2D analysis curve for the relative yield per recruit and the relative biomass per recruit of *P. trituberculatus* based on different natural mortality (*M*) estimation methods. (**a**) *M* = 0.39; (**b**) *M* = 0.65; (**c**) *M* = 1; (**d**) *M* = 0.86; (**e**) *M* = *M_mean_* = 0.7.

**Figure 9:**
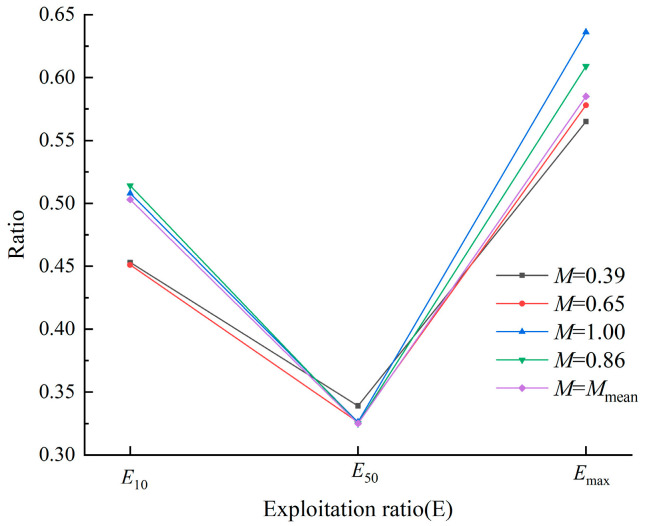
Changes in resource utilization rates at *E*_10_, *E*_50_, and *E_max_* levels based on different natural mortality (*M*).

Based on the three-year biological parameters of *P. trituberculatus* and the *M* values estimated using different empirical formulas (*M* = 0.39, 0.65, 1.00, 0.86), as well as their mean reference value (*M_mean_* = 0.70), a yield per recruit model for the total population of *P. trituberculatus* was established (Figure 8). Under a given total mortality coefficient, the relative yield per recruit of the total population gradually decreased with increasing *M*. On this basis, the resource utilization status of *P. trituberculatus* was examined when the population biomass declined to 10% and 50% of the unexploited level and when the maximum yield was achieved (Figure 9). The results showed that when *M* was at its minimum value, the exploitation rate at 50% of the unexploited biomass (*E*_50_) was 0.339, which was higher than under other *M* scenarios, where *E*_50_ remained around 0.326. As *M* increased, the exploitation rates at 10% of the unexploited biomass (*E*_10_) and at maximum sustainable yield (*E_max_*) increased accordingly. Based on the mean reference value (*M_mean_* = 0.70), the exploitation rates for the total population of *P. trituberculatus* were *E*_10_ = 0.503, *E*_50_ = 0.325, and *E_max_* = 0.585.

## 4. Discussion

### 4.1. Growth Status of P. trituberculatus in Laizhou Bay

Understanding the growth status of *P. trituberculatus* provides a theoretical basis for the rational utilization of its resources. In this study, the carapace width–body weight relationships for *P. trituberculatus* in Laizhou Bay were *W* = 7.145 × 10^−5^*L*^2.9153^ for the total population, *W* = 1.208 × 10^−4^*L*^2.8051^ for females, and *W* = 3.209 × 10^−5^*L*^3.0809^ for males. The female-to-male sex ratio was 0.99, with males being slightly dominant. Generally, the parameter *a* in the carapace width–body weight relationship reflects the environmental conditions of the population [21], and favorable environmental conditions such as suitable hydrological regimes and abundant prey availability can lead to an increase in parameter *a*. In Laizhou Bay, long-term monitoring records indicate that the annual mean water temperature has remained relatively stable at approximately 12 °C over the past decade, with seasonal fluctuations following typical temperate patterns [13]. Food availability for benthic predators has likely declined due to intensified resource competition from expanding shellfish aquaculture and deteriorating habitat quality [3]. Given the relatively stable thermal regime, the observed differences in parameter *a* compared with historical data are more plausibly attributed to changes in prey availability and interspecific competition rather than to temperature shifts. The power exponent *b* typically reflects the rate at which body weight increases with carapace width. In this study, the *b* value of male *P. trituberculatus* was higher than that of females, indicating a slightly faster increase in body weight relative to carapace width in males [22]. The power exponent *b* is also used as an indicator of isometric growth in swimming crabs [23]. Studies have shown that *b* values generally range between 2.5 and 3.5 [24], with *b* = 3 representing ideal isometric growth, meaning that the growth rates in length, width, and height remain consistent throughout the individual’s life [19].

In this study, the estimated *b* values for *P. trituberculatus* were 2.9153, 2.8051, and 3.0809, indicating near-isometric growth in males and negative allometric growth in females. Using the ELEFAN method, the asymptotic carapace widths (*L*_∞_) for the total, female, and male populations were estimated as 228.32 mm, 227.23 mm, and 215.25 mm, respectively. Compared with data from a decade ago, the asymptotic carapace width of females in Laizhou Bay decreased by 6%, while that of males increased by 2.5%. During the spring–summer season (April to August), ovigerous females often inhabit shallow waters (3–5 m depth) within the bay, making them more vulnerable to capture [3]. Additionally, market demand for mature females has led to selective fishing pressure on large females. Consequently, competition pressure on males for food and space has decreased, and niche release has contributed to an increase in the asymptotic carapace width of males.

The growth coefficients (*k*) for female and male crabs were 0.49 and 0.44, respectively, and the overall *k* value for the total population was only 0.43. Compared with previous studies in Laizhou Bay, the *k* value of *P. trituberculatus* has significantly decreased [7], and it is also lower than the results reported for the coastal waters of Qingdao [25] and Zhejiang [26] (Table 3). Although the sample size of *P. trituberculatus* in this study (2240 ind.) was larger than that of previous studies in Laizhou Bay, the marked decline in *k* values may reflect a population decline in this area between 2012 and 2023–2025. In recent years, the population size of *P. trituberculatus* in Laizhou Bay has been slightly restored through systematic stock enhancement programs; however, the resource level remains below historical peaks [27]. Currently, the Laizhou Bay ecosystem is dominated by benthic organisms, with a relatively simple food web structure [28]. Cultured shellfish exhibit a certain degree of habitat overlap with *P. trituberculatus*. The increase in scallop farming density during 2021–2022 has further intensified food competition within the bay [29]. Under conditions of limited prey availability for *P. trituberculatus*, large-scale stock enhancement activities may further deteriorate the feeding conditions of wild populations, thereby failing to achieve the desired resource restoration outcomes [30]. Reduced prey availability can decrease the growth rate of *P. trituberculatus* [31]; not only that, but environmental pollution caused by coastal development has led to a decline in water quality in Laizhou Bay [32]. For instance, elevated dissolved inorganic nitrogen (DIN) and persistently high N/P ratios indicate phosphorus-limited eutrophication. Long-term monitoring has shown that although dissolved oxygen and chemical oxygen demand generally meet the Class I seawater quality standards, DIN pollution is severe, with up to 31% of survey stations exceeding the Class IV standard [13]. Since 2004, the trophic conditions of the bay have been poor, with DIN identified as the primary driver of eutrophication (contributing 21.4% to the comprehensive eutrophication index) [33]. Prolonged exposure to such nutrient-enriched environments may impose physiological stress on benthic crustaceans, potentially affecting their molting frequency, metabolic efficiency, and overall growth [4], which may also reduce the growth performance of the species.

Across different sea regions, variations in natural conditions such as temperature, salinity, prey availability, and hydrological environment often lead to distinct biological characteristics in *P. trituberculatus* populations. These inter-regional isolation mechanisms may drive independent differentiation of local populations [34], ultimately forming groups with unique growth characteristics. The observed differences in growth parameters may be attributed to regional characteristics of the aquatic environment. Taking the coastal waters of Laizhou Bay as an example, water temperature conditions and food resources collectively constrain the growth rate of *P. trituberculatus* in this area.

**Table 3:** Biological parameters of *P. trituberculatus* in different sea areas.

Area	Gender	*a*	*b*	*L*_∞_/mm	*k*/a^−1^	*M*/a^−1^	*Z*/a^−1^	*F*/a^−1^	*E*	Reference
Laizhou Bay (2023~2025)	Female	1.21 × 10^−4^	2.81	227.23	0.49	0.87	1.44	0.57	0.40	this study
	Male	3.21 × 10^−5^	3.08	215.25	0.44	0.71	1.30	0.59	0.45	
	Total	7.15 × 10^−5^	2.92	228.32	0.43	0.70	1.41	0.71	0.50	
Laizhou Bay (2012)	Female	5.81 × 10^−5^	2.96	241.50	1.60	1.20	4.17	2.97	0.71	[7]
	Male	7.75 × 10^−5^	2.90	210.00	1.50	1.19	3.76	2.57	0.68	
Qingdao (2018~2022)	Female	0.46	2.93	215.00	0.78	1.20	1.80	0.60	0.33	[25]
	Male	0.46	2.87	209.40	0.75		2.18	0.98	0.45	
	Total	0.45	2.91	213.00	0.75		2.08	0.88	0.42	
Zhejiang (2015–2016)	Female	0.07	2.88	210.00	0.97	1.71	2.94	1.23	0.42	[35]
	Male	0.06	2.94		0.72					
	Total	0.06	2.91		0.83					

### 4.2. Resource Utilization Status of P. trituberculatus

The bimodal recruitment pattern observed in male *P. trituberculatus* during June and July is most likely driven by divergent survival patterns and asynchronous molting phenology. In Laizhou Bay, hatchery-released juveniles are typically stocked from late May to late June. Females generally molt earlier than males, which tend to delay molting until July, when water temperatures reach 20–23 °C, the optimal range for post-molt recovery and growth [31]. This sex-asynchronous molting schedule generates a two-pulse structure: an early pulse in June from females and a later pulse in July from males. Temperature also influences larval development and metamorphosis [36], with warmer spring conditions favoring early cohorts, while suboptimal temperatures later in the season may reduce late-larval survival [37]. Notably, this bimodality was evident only in recruitment magnitude, while the overall seasonal recruitment pattern remained continuous. These findings emphasize the need to account for sex-specific life histories and thermal influences when interpreting recruitment and planning stock enhancement.

In this study, the estimated natural mortality coefficients (*M*) for *P. trituberculatus* in Laizhou Bay from 2023 to 2025 were *M* = 0.70 for the total population, *M* = 0.87 for females, and *M* = 0.71 for males. Compared with the survey conducted in Laizhou Bay in 2012 [38], the estimated *M* values have decreased, a trend directly linked to the population’s growth rate. Populations with slower growth rates generally exhibit lower natural mortality. From a biological perspective, slower growth may reflect a lower metabolic rate, thereby reducing mortality risks associated with energy expenditure, activity-related exposure, and predation pressure. Furthermore, the *M*/*k* ratios estimated in this study ranged from 1.61 to 1.78, all falling within the range of 1.5 to 2.5 proposed by Beverton & Holt [39]. In terms of resource utilization, the optimal exploitation rate for the population is 0.5. This study found that the exploitation rate at which the maximum yield was achieved was 0.585, which is close to the optimal exploitation rate. Additionally, the exploitation rate of the population during 2023–2025 ranged from 0.40 to 0.50, which is lower than both the optimal exploitation rate and the theoretical exploitation rate at the maximum yield.

Compared with the population exploitation rate in 2012 (0.684–0.712), the current exploitation rate has significantly decreased. Along with a concurrent decline in the fishing mortality coefficient, this indicates that the *P. trituberculatus* resource in Laizhou Bay has gradually recovered from a severely overexploited status [7,9,34] to a reasonably utilized level. The main recruitment period of *P. trituberculatus* from April to August coincides with both its reproductive biology and the summer fishing moratorium in Laizhou Bay. During this moratorium period, which aligns with the larval hatching period [40] and the stock enhancement season of *P. trituberculatus*. Stock enhancement activities not only directly increase the recruitment of the current year but may also contribute to the recruitment of the following year through the survival of overwintering individuals. The fishing ban provides protection for both natural and hatchery-released juveniles, allowing gradual resource replenishment and promoting the recovery of *P. trituberculatus* resources in this area [41]. The decline in the exploitation rate (*E*) to a reasonably utilized level during 2023–2025 reflects decreasing fishing pressure over the years. However, the observed declines in asymptotic carapace width (*L*_∞_) and growth coefficient (*k*) still reflect the lag effects of long-term overfishing.

### 4.3. Potential Mechanisms Underlying Growth Decline

Historical fishing practices in Laizhou Bay, particularly the sustained targeting of larger individuals, may have imposed strong directional selection on life-history traits of *P. trituberculatus*. Under such size-selective mortality, populations often exhibit evolutionary shifts toward slower growth rates and earlier maturation at smaller body sizes [42], as individuals with faster growth and larger asymptotic size are disproportionately removed prior to reproduction [43]. This phenomenon has been documented in harvested fish populations and analogous to observed patterns in exploited crustaceans, where prolonged size-selective harvesting led to reduced asymptotic size and altered growth trajectories [44,45]. The marked decline in the growth coefficient (*k*) observed in this study, from 1.50 to 1.60 in 2012 to 0.43 to 0.49 in 2023–2025, together with the concurrent reduction in asymptotic carapace width (*L*_∞_), is broadly consistent with the predicted outcomes of long-term size-selective mortality. Although direct genetic evidence is lacking in the present study, this evolutionary hypothesis warrants rigorous testing through genomic approaches and quantitative genetic analyses in future investigations.

On the other hand, Shandong Province has implemented a large-scale stock enhancement program for *P. trituberculatus* in Laizhou Bay since 2005, with tens of millions of juveniles released annually [9]. Although these releases contribute to population replenishment, the sustained high-density stocking may induce negative density-dependent effects on individual growth. When population density exceeds the carrying capacity of the ecosystem, intensified intraspecific competition for limited food and spatial resources can reduce growth rates and average body size. This mechanism is particularly relevant for benthic predators such as *P. trituberculatus*, whose food resources (bivalves, small crustaceans, and polychaetes) are spatially limited and prone to depletion. Previous Ecopath modeling studies have estimated the ecological carrying capacity for *P. trituberculatus* in Laizhou Bay at approximately 1.107 t·km^−2^ [27]. More recent assessments that account for food competition from shellfish aquaculture have yielded lower estimates, ranging from 0.547 to 0.567 t·km^−2^ [3]. These values suggest a limited capacity for further biomass increases within the bay. The observed decline in growth parameters, despite reduced fishing pressure, may therefore reflect density-dependent growth suppression resulting from sustained high-density stocking. This interpretation is further corroborated by the findings of Fu et al. [25], who reported that the growth coefficient (*k*) of *P. trituberculatus* in Qingdao coastal waters (0.75–0.78) was markedly lower than that observed in Laizhou Bay during 2012 (1.50–1.60), suggesting a broader regional trend of growth suppression potentially linked to high-density stocking practices.

### 4.4. Limitations and Prospects

This study used the von Bertalanffy growth function (VBGF) to model the growth of *P. trituberculatus*. This method was originally developed for fish with continuous growth [10,46], whereas crustaceans completely shed their hard exoskeleton during molting [47], exhibiting a discontinuous, stepwise growth pattern. Theoretically, VBGF is not the optimal model for describing crustacean growth. However, Bergman et al. argued that molting among individuals in a reproductive population does not occur synchronously, and the overall growth process can still be considered continuous [48]. *P. trituberculatus* inhabiting the same sea area are influenced by similar environmental conditions, and their biological characteristics exhibit relative stability [35]. In this study, the VBGF was fitted and growth parameter curves were generated using the FiSAT II software, which objectively reflected characteristics such as fast growth and a short life cycle. However, the approach was relatively simplistic, as it did not incorporate a seasonal VBGF for growth assessment. Given that the growth of *P. trituberculatus* often exhibits seasonal fluctuations, the omission of a seasonal VBGF may not adequately address the potential issue of negative growth within the population [49]. In future studies, in addition to evaluating seasonal VBGF growth of *P. trituberculatus* in Laizhou Bay, the influence of alternative growth models (e.g., Gompertz and logistic growth equations) on assessment results should also be explored.

The natural mortality coefficient (*M*) is one of the most important parameters in population resource assessment and management [50]; however, it is often difficult to obtain directly. Previous studies have mostly relied on empirical methods to estimate *M* [51]. It should be noted that these empirical formulas were originally developed for fish, and their applicability to crustaceans may be constrained by species-specific biological characteristics. In this study, eight commonly used empirical formulas were applied to estimate *M* for *P. trituberculatus*, yielding a relatively wide range of 0.39–1.00. Although averaging multiple estimates can reduce uncertainty to some extent [52], the selection of formulas may still introduce biases into the results. Future studies should incorporate mark-recapture methods to improve the understanding of life history traits of the released population, including growth, mortality, and reproduction, as well as to evaluate temporal changes in *M*, thereby enhancing the accuracy of resource assessments.

Distinguishing between evolutionary and density-dependent mechanisms is critical for adaptive management. If fisheries-induced evolution is the primary driver, recovery would require a multi-generational relaxation of selective pressure, potentially through increased minimum capture size and protection of large individuals. If density-dependent competition dominates, management should focus on optimizing release numbers and evaluating the ecological carrying capacity under current environmental conditions. This study focused on a single-species assessment, whereas interactions between the natural environment and predators may also influence natural mortality. *P. trituberculatus* occupies mid-to-upper trophic levels and changes in its population may affect the population dynamics of other species through the food web [53]. Conversely, variations in prey abundance may affect the survival of *P. trituberculatus*, which in turn underscores the need for ecosystem-based approaches. Future research should develop ecosystem models (e.g., Ecopath with Ecosim) to conduct long-term dynamic simulations of the Laizhou Bay ecosystem, thereby better capturing changes in the mortality of *P. trituberculatus* in response to both biotic and abiotic environmental factors. Ecosystem modeling will be essential to disentangle these mechanisms and guide sustainable stock-enhancement strategies in Laizhou Bay.

## 5. Conclusions

This study provides an updated assessment of the growth, mortality, and stock status of *P. trituberculatus* in Laizhou Bay based on 2023–2025 survey data. The exploitation rate has declined from severely overexploited levels (0.684–0.712 in 2012) to around the MSY reference point (*E* = 0.50), yet key biological parameters have continued to deteriorate over the same period. The growth coefficient has dropped by approximately 70% (from 1.50 to 1.60 to 0.43–0.49), and the asymptotic carapace width has also decreased. This decoupling between reduced fishing pressure and declining population productivity suggests that historical overexploitation, compounded by intensifying food competition from shellfish aquaculture and deteriorating water quality, has left the stock in a vulnerable state with no signs of fundamental recovery despite sustained management interventions.

These findings carry several immediate management implications. First, increasing the current minimum capture size to protect juvenile individuals of the population. Second, given the substantial decline in growth rates, increasing the trawl codend mesh size from 20 mm to 30–35 mm to reduce the capture of slow-growing individuals. Third, core juvenile grounds should be designated as permanent or seasonal no-take zones. The implementation of these measures should be coupled with targeted research to enable adaptive refinement. Extended length–frequency monitoring and mark-recapture studies can directly validate the effectiveness of size and mesh adjustments. Ecosystem models can dynamically guide spatial and temporal closures by evaluating how food competition and habitat changes affect carrying capacity and recruitment success. Genetic monitoring will serve as an early-warning system for detecting loss of adaptive diversity. This integrated feedback loop grounds management actions in current data while refining them through scientific evidence, providing a robust basis for sustainable stock enhancement and fisheries’ management in Laizhou Bay.

## Figures and Tables

**Table 1:** 8 Common methods to estimate natural mortality (*M*).

Method	Equation	Parameter	Reference
1	$\mathrm{lgM}=-0.0066-0.2790\mathrm{lg}L_{\infty }+0.6543\mathrm{lgK}+0.4634\mathrm{lgT}$	*L*_∞_ = 228.32 *k* = 0.43, *T* = 12	[11]
2	$\mathrm{lgM}=-0.2107-0.0824\mathrm{lg}W_{\infty }+0.6757\mathrm{lgK}+0.4627\mathrm{lgT}$	*W*_∞_ = 537.27 *k* = 0.43, *T* = 12	[11]
3	$M=e^{1.44-0.982\ln T_{\max}}$	*T_max_* = 3	[15]
4	M=1.5K	*k* = 0.43	[16]
5	$M=-\mathrm{lnP}/T_{\max}$	*P* = 0.05, *T_max_* = 3	[17]
6	$M=4.31{\left(t_{0}-\frac{\ln0.05}{K}\;\right)}^{-1.01}$	*t*_0_ = −0.46, *k* = 0.43	[18]
7	$M=-0.0021+2.5912/T_{\max}$	*T_max_* = 3	[19]
8	$M\;=3K/\left(e^{0.38KT_{\max}}-1\right)$	*k* = 0.43, *T_max_* = 3	[20]
9	M= *M_mean_*	–	–

Note: *L*_∞_ indicates asymptotic length (mm), *K* indicates growth coefficient (/a), *T* indicates temperature (°C), *W*_∞_ indicates asymptotic weight (g), *T_max_* indicates maximum age, *P* is the proportion surviving to maximum age, *t*_0_ is the theory age at zero length, and *M_mean_* indicates the mean value of eight natural mortality.

**Table 2:** Results of natural mortality (*M*) based on different estimation methods.

Sampling	Method	*M*/a^−1^	*F*/a^−1^	*E*	*M*/*K*
Total	1	0.39	1.02	0.72	0.91
	2	0.65	0.76	0.54	1.51
	3	0.65	0.76	0.54	1.51
	4	1.00	0.41	0.29	2.33
	5	0.65	0.76	0.54	1.51
	6	0.86	0.55	0.39	2.00
	7 *	0.70	0.71	0.50	1.63
Female	1	0.43	1.01	0.70	0.88
	2	0.72	0.72	0.50	1.47
	3	0.74	0.70	0.49	1.51
	4	1.00	0.44	0.31	2.04
	5	0.74	0.70	0.49	1.51
	6	0.86	0.58	0.40	1.76
	7 *	0.87	0.57	0.40	1.78
Male	1	0.41	0.89	0.68	0.93
	2	0.66	0.64	0.49	1.50
	3	0.67	0.63	0.48	1.52
	4	1.00	0.30	0.23	2.27
	5	0.67	0.63	0.48	1.52
	6	0.86	0.44	0.34	1.95
	7 *	0.71	0.59	0.45	1.61

Note: * indicates the mean value of natural mortality (*M_mean_*).

## Data Availability

Dataset available on request from the authors.

## References

[1] Song P. (1982). Morphology and habits of the swimming crab *Portunus trituberculatus*. Bull. Biol..

[2] Chen D., Shen W., Liu Q., Jiao Y., Zeng X., Ren Y. (2000). The geographical characteristics and fish species diversity in the Laizhou Bay and Yellow River estuary. J. Fish. Sci. China.

[3] Chen S., Zhang S., Wang X., Li F., Zhang X. (2025). Impacts of shellfish aquaculture on the ecological carrying capacity of *Portunus trituberculatus* in Laizhou bay. Aquac. Int..

[4] Shan X. (2013). Long-Term Changes in Fish Assemblage Structure in the Yellow River Estuary Ecosystem, China. Mar. Coast. Fish..

[5] Zhang X., Wang X., Tu Z., Zhang P., Wang Y., Gao T., Wang S. (2009). Current status and prospect of fisheries resource enhancement in Shandong Province. Chin. Fish. Econ..

[6] Zhang B., Jin X., Wu Q., Xie Z. (2015). Enhancement and release of Chinese shrimp in Laizhou Bay. J. Fish. Sci. China.

[7] Yang G., Xu B., Wang X., Li F., Yuan X., Lv Z. (2017). On biological parameters and growth characteristics of *Portunus trituberculatus* in the Laizhou Bay. Mar. Fish..

[8] Wu Q., Wang J., Chen R., Huang J., Zuo T., Luan Q., Jin X. (2016). Biological characteristics, temporal-spatial distribution of *Portunus trituberculatus* and relationships between its density and impact factors in Laizhou Bay, Bohai Sea, China. Chin. J. Appl. Ecol..

[9] Cong X. (2015). Study on the Crab Community Structure in Laizhou Bay and the Trophic Niche of *Portunus trituberculatus*. Master’s Thesis.

[10] Chen G., Li Y., Chen P., Shu L. (2008). Optimum interval class size of length-frequency analysis of fish. J. Fish. Sci. China.

[11] Pauly D. (1980). On the interrelationships between natural mortality, growth parameters, and mean environmental temperature in 175 fish stocks. ICES J. Mar. Sci..

[12] Brodziak J., Ianelli J., Lorenzen K., Methot R. (2011). Estimating Natural Mortality in Stock Assessment Applications.

[13] Zhao J., Chen J. (2001). Research on aquacultural hydro-environment of Eastern Laizhou Bay. Mar. Fish. Res..

[14] Dai A., Feng Z., Song Y., Huang Z., Wu H. (1977). A preliminary investigation on the fishery biology of *Portunus trituberculatus*. Chin. J. Zool..

[15] Hoenig J.M. (1983). Empirical use of longevity data to estimate mortality rates. Fish. Bull..

[16] Jensen A.L. (1996). Beverton and holt life history invariants result from optimal trade-off of reproduction and survival. Can. J. Fish. Aquat. Sci..

[17] Tanaka S. (1960). Studies on the dynamics and management of fish populations. Bull. Tokai Reg. Fish. Res. Lab..

[18] Cubillos L.A., Alarcón R., Brante A. (1999). Empirical estimates of natural mortality for the chilean hake (*Merluccius gayi*): Evaluation of precision. Fish. Res..

[19] Zhan B. (1995). Fishery Resource Assessment.

[20] Alverson D.L., Carney M.J. (1975). A graphic review of the growth and decay of population cohorts. ICES J. Mar. Sci..

[21] Lin X. (1999). Studies on the length-weight relationship of male *Upeneus bensari* in coastal waters of Eastern Guangdong. J. Shantou Univ. (Nat. Sci.).

[22] Guan W., Xuan F., Chen H., Dai X., Zhu J. (2009). Reproductive characteristics and condition status of adult swimming crab *Portunus trituberculatus* (Brachyura: Portunidae) in East China Sea. Mar. Fish..

[23] Wang X., Wang Y., Ye T., Lu W., Zhou C. (2018). A Preliminary Analysis of the Growth Characteristics of *Portunus trituberculatus*. Trans. Oceanol. Limnol..

[24] Atar H.H., Secer S. (2003). Width/length-weight relationships of the blue crab (*Callinectes sapidus* rathbun 1896) population living in beymelek lagoon lake. Turk. J. Vet. Anim. Sci..

[25] Fu D., Zhao T., Zhang C., Ren Y. (2026). Evaluating stock enhancement effects of *Portunus trituberculatus* with respect to the biological differences between males and females. J. Fish. Sci. China.

[26] Ye T. (2017). Analysis of Biological Characteristics of *Portunus trituberculatus* and the Determination of Releasing Capacity. Master’s Thesis.

[27] Zhang M., Leng Y., Lv Z., Li F., Wang T., Zhang A. (2013). Estimating the ecological carrying capacity of *Portunus trituberculatus* in Laizhou Bay. Mar. Fish..

[28] Zhang B., Wu Q., Jin X. (2015). Interannual variation in the food web of commercially harvested species in Laizhou Bay from 1959 to 2011. J. Fish. Sci. China.

[29] Zhang J., Fang J., Wang W. (2009). Progress in studies on ecological carrying capacity of mariculture for filter-feeding shellfish. J. Fish. Sci. China.

[30] Smith J.A., Baumgartner L.J., Suthers I.M., Fielder D.S., Taylor M.D. (2013). Density-dependent energy use contributes to the self-thinning relationship of cohorts. Am. Nat..

[31] Liao Y., Xiao Z., Yuan Y. (2008). Temperature tolerance of larva and juvenile of *Portunus trituberculatus*. Acta Hydrobiol. Sin..

[32] Liu X., Zhang J., Zhao Y., Qiao L., Li M., Yang Y., Sun S., Li K., Wang X. (2025). An improved composite eutrophication index for coastal assessment: Laizhou Bay, China. Mar. Environ. Res..

[33] Liao Y., Chen R. (2007). The effect of sublethal concentrations of Cu, Pb, Cd and Hg on larval development of *Portunus trituberculatus*. J. Environ. Sci..

[34] Liu S., Xue S., Sun J. (2008). Genetic diversity of *Portunus triuberbuculatus* revealed by aflp analysis. Oceanol. Limnol. Sin..

[35] Wang X. (2017). Growth Characteristics of *Portunus trituberculatus* in Zhejiang Fishing Ground. Master’s Thesis.

[36] Wang H., Gao B., Duan Y., Han X., Liu P., Li J. (2014). Comparison of reproduction and early growth of offsprings betweenhybrid and inbred families in swimming crab *Portunus trituberculatus*. J. Dalian Ocean Univ..

[37] Zhu K., Li Z., Zhou Y., Xu K., Zhu W., Wang Z. (2021). Growth, mortality parameters and exploitation of the swimming crab *Portunus trituberculatus* (Miers, 1876) in the East China Sea. Indian J. Fish..

[38] Yang G. (2017). On Community Structure of Crab and Biological Parameters and Stock Number of *Portunus trituberculatus* in Shandong Offshore. Master’s Thesis.

[39] Beverton R.J.H., Holt S.J. (1959). A review of the lifespans and mortality rates of fish in nature, and their relation to growth and other physiological characteristics. Ciba Foundation Symposium-the Lifespan of Animals (Colloquia on Ageing).

[40] Jia L. (2009). Preliminary Study on the Ovary Development of the Crab *Portunus trituberculatus*. Master’s Thesis.

[41] Liu L., Wan R., Duan Y., Wang X., Wang S., Wang Y. (2008). Status and effect of enhancement release of marine fisheries resource in Shandong. Trans. Oceanol. Limnol..

[42] Law R. (2000). Fishing, selection, and phenotypic evolution. ICES J. Mar. Sci..

[43] Jørgensen C., Enberg K., Dunlop E.S., Arlinghaus R., Boukal D.S., Brander K., Ernande B., Gårdmark A., Johnston F., Matsumura S. (2007). Managing Evolving Fish Stocks. Science.

[44] Edeline E., Carlson S.M., Stige L.C., Winfield I.J., Fletcher J.M., James J.B., Haugen T.O., Vøllestad A.L., Stenseth N.C. (2007). Trait changes in a harvested population are driven by a dynamic tug-of-war between natural and harvest selection. Proc. Natl. Acad. Sci. USA.

[45] Uusi-Heikkilä S., Wolter C., Klefoth T., Arlinghaus R. (2008). A behavioral perspective on fishing-induced evolution. Trends Ecol. Evol..

[46] Ye T., Wang Y., Zhou C. (2014). Analysis of effects of fish length frequency data on estimates of growth parameters. Fish. Sci..

[47] Penn J. (1980). Spawning and fecundity of the western king prawn, *Penaeus latisulcatus* kishinouye, in western australian waters. Aust. J. Mar. Freshw. Res..

[48] Bergman P.K., Haw F., Blankenship L.H., Buckley R.M. (1992). Perspectives on design, use, and misuse of fish tags. Fisheries.

[49] Li Z. (2006). The Study on the Performances of Length-Frequency Analysis Methods on the Simulated and Real Fishery Data Sets. Ph.D. Thesis.

[50] Lee H.H., Maunder M.N., Piner K.R., Methot R.D. (2011). Estimating natural mortality within a fisheries stock assessment model: An evaluation using simulation analysis based on twelve stock assessments. Fish. Res..

[51] Vetter E. (1988). Estimation of natural mortality in fish stocks: A review. Fish. Bull..

[52] Hewit D.A., Lambert D.M., Hoenig J.M., Lipcius R.N., Bunnell D.B., Miller T.J. (2007). Direct and indirect estimates of natural mortality for chesapeake bay blue crab. Trans. Am. Fish. Soc..

[53] Lin Q., Yuan W., Ma Y., Zu K., Zhang H. (2022). Evaluation of project to enhance the ecological carrying capacity of swimming crab (*Portunus trituberculatus*) in haizhou bay. J. Hydroecol..

